# Weldable and closed-loop recyclable monolithic dynamic covalent polymer aerogels

**DOI:** 10.1093/nsr/nwac012

**Published:** 2022-01-28

**Authors:** Xinhai Zhang, Jun Zhao, Kai Liu, Guangfeng Li, Dong Zhao, Zhaoming Zhang, Junjun Wan, Xue Yang, Ruixue Bai, Yongming Wang, Wei Zhang, Xuzhou Yan

**Affiliations:** School of Chemistry and Chemical Engineering, Frontiers Science Center for Transformative Molecules, Shanghai Key Laboratory of Electrical Insulation and Thermal Aging, Shanghai Jiao Tong University, Shanghai 200240, China; School of Chemistry and Chemical Engineering, Frontiers Science Center for Transformative Molecules, Shanghai Key Laboratory of Electrical Insulation and Thermal Aging, Shanghai Jiao Tong University, Shanghai 200240, China; School of Chemistry and Chemical Engineering, Frontiers Science Center for Transformative Molecules, Shanghai Key Laboratory of Electrical Insulation and Thermal Aging, Shanghai Jiao Tong University, Shanghai 200240, China; School of Chemistry and Chemical Engineering, Frontiers Science Center for Transformative Molecules, Shanghai Key Laboratory of Electrical Insulation and Thermal Aging, Shanghai Jiao Tong University, Shanghai 200240, China; School of Chemistry and Chemical Engineering, Frontiers Science Center for Transformative Molecules, Shanghai Key Laboratory of Electrical Insulation and Thermal Aging, Shanghai Jiao Tong University, Shanghai 200240, China; School of Chemistry and Chemical Engineering, Frontiers Science Center for Transformative Molecules, Shanghai Key Laboratory of Electrical Insulation and Thermal Aging, Shanghai Jiao Tong University, Shanghai 200240, China; School of Chemistry and Chemical Engineering, Frontiers Science Center for Transformative Molecules, Shanghai Key Laboratory of Electrical Insulation and Thermal Aging, Shanghai Jiao Tong University, Shanghai 200240, China; School of Chemistry and Chemical Engineering, Frontiers Science Center for Transformative Molecules, Shanghai Key Laboratory of Electrical Insulation and Thermal Aging, Shanghai Jiao Tong University, Shanghai 200240, China; School of Chemistry and Chemical Engineering, Frontiers Science Center for Transformative Molecules, Shanghai Key Laboratory of Electrical Insulation and Thermal Aging, Shanghai Jiao Tong University, Shanghai 200240, China; School of Chemistry and Chemical Engineering, Frontiers Science Center for Transformative Molecules, Shanghai Key Laboratory of Electrical Insulation and Thermal Aging, Shanghai Jiao Tong University, Shanghai 200240, China; Department of Chemistry, University of Colorado Boulder, Boulder, CO 80309, USA; School of Chemistry and Chemical Engineering, Frontiers Science Center for Transformative Molecules, Shanghai Key Laboratory of Electrical Insulation and Thermal Aging, Shanghai Jiao Tong University, Shanghai 200240, China

**Keywords:** dynamic covalent polymer, aerogel, weldability, degradability, closed-loop recyclability

## Abstract

Owing to their low density, high porosity and unique micro-nanostructures, aerogels are attractive for application in various fields; however, they suffer from shrinkage and/or cracking during preparation, mechanical brittleness, low production efficiency and non-degradation. Herein, we introduce the concept of dynamic covalent polymer chemistry to produce a new class of aerogels—referred to as DCPAs. The resulting lightweight DCPAs have the potential to be prepared on a large scale and feature high porosity (90.7%–91.3%), large degrees of compression (80% strain) and bending (diametral deflection of 30 mm) without any cracks, as well as considerable tensile properties (an elongation with a break at 32.7%). In addition, the DCPAs showcase the emergent characteristics of weldability, repairability, degradability and closed-loop recyclability that are highly desirable for providing versatile material platforms, though hardly achieved by traditional aerogels. Taking advantage of their robust porous structures, we demonstrate the potential of DCPAs for applications in thermal insulation and emulsion separation. These findings reveal that the dynamic covalent bond strategy would be generalized for the production of a new generation of aerogels with customized features for functioning in the field of intelligent and sustainable materials.

## Teaser

Dynamic covalent polymer aerogels (DCPAs) are constructed for the first time. It represents a new concept of aerogels with emerging characteristics of weldability, repairability, and closed-loop recyclability.

## INTRODUCTION

Weight reduction, performance improvement, cost reduction and sustainability have been the most significant drives in the design of structural materials [1,2]. Aerogels, which are porous sol–gel materials, have been recognized as pivotal components for advanced structural materials due to their fascinating characteristics of low density (0.001–0.3 g/cm^3^) and high porosity (>80%) [3–5]. They have been widely utilized in various fields, such as energy storage [6], drug delivery [7], sensors [8], sound absorption [9], electromagnetic shielding [10], thermal insulation [5] and water treatment [11]. However, the inevitable shrinkage and/or cracking induced by capillary force during the drying process together with the brittle nature of most aerogels themselves, have been long-standing and intractable issues [12]. To fabricate monolithic aerogels with desired performance, special drying methods that can decrease or eliminate the undesirable capillary force, such as supercritical drying and freeze-drying, are always considered; however, such methods require specialized drying devices that result in a low production rate and high cost. Therefore, it is of great necessity and importance to solve the above-mentioned issues and simultaneously endow the aerogels with emergent features and functionalities.

Dynamic covalent polymer networks (DCPNs) [13,14], which possess the benefits of thermosets yet retain reprocessability resembling thermoplastics, have been widely explored in synthetic chemistry and materials science [15–22]. Under certain conditions, the dynamic networks remain stable, though polymer chains could depolymerize, or rearrangement of the topological network structures could occur once the dynamic covalent bonds are activated [23–26]. For the construction of aerogels, DCPNs would be great candidates when considering the following merits: (i) The dynamic nature of DCPNs would endow the aerogels with repairability and chemical recyclability, which are scarcely attainable by traditional aerogels (e.g. inorganic silica aerogels and organic resorcinol–formaldehyde aerogels); (ii) The reversible cross-linked DCPNs are able to generate robust skeletons that would prevent gel shrinkage and/or cracking during the drying process and simultaneously ensure the mechanical performance of the resultant aerogels; (iii) The family of aerogels would be significantly enriched due to the variety of DCPNs. As such, we envision that DCPNs will be able to take the development of aerogels to the next level of simplicity, practicality and sustainability. Nevertheless, dynamic covalent polymer chemistry has remained unexploited in the fabrication of monolithic aerogels.

Herein, we present a straightforward one-pot, mild and catalyst-free polycondensation strategy via dynamic imine chemistry, with the combination of low-cost and promising ambient pressure drying, to construct monolithic dynamic covalent polymer aerogels (DCPAs). The chemistry involved is simple and practical. Specifically, we first synthesized a polyimine oligomer using terephthalaldehyde (TA) and diethylenetriamine (DETA). Subsequently, tris(2-aminoethyl)amine (TREN) as a cross linker was applied to induce the formation of highly cross-linked but dynamic polyimine gels. After solvent exchanges and ambient pressure drying, the resulting DCPAs exhibited low shrinkage that was comparable to that of the aerogels prepared by supercritical drying and freeze-drying. Benefiting from the reversible dynamic covalent bonds, as well as the robust gel skeletons, the DCPAs simultaneously exhibit flexibility, weldability and repairability. Moreover, the DCPAs could be readily depolymerized into soluble oligomers and/or monomers by introducing an excess of free amine groups. Impressively, new monolithic aerogels could be regenerated from the recyclable solution after the free amine groups were consumed, thereby achieving a closed-loop chemical recyclability. Finally, we exploited the porous structures and robust skeletons to demonstrate the multifunctionality of the DCPAs, in areas such as thermal insulation and emulsion separation.

## RESULTS AND DISCUSSION

### Design, fabrication, structural characterization and basic properties of the DCPAs

To improve the fabrication of the DCPAs, we abide by three criteria: (i) the preparation process should be simple and suitable for large-scale production; (ii) the lightweight DCPAs should possess qualified mechanical properties to enhance practicability; (iii) the skeletons of the DCPAs should contain abundant dynamic bonds to realize chemical recyclability. To meet the first requirement, ambient pressure drying, which avoids the use of special equipment such as a supercritical dryer or a freeze dryer, is adopted. Polyimine, prepared from commercially available dialdehyde, diamine and triamine with a readily controllable polycondensation process, represents an ideal candidate. On the one hand, the high cross-linking density can ensure the mechanical property requirement of the second criterion. On the other hand, plenty of reversible imine bonds are able to satisfy the third requirement.

**Figure 1.**
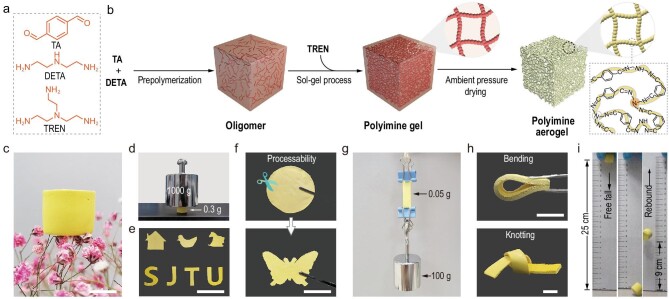
Illustrations of the preparation and performances of DCPAs. (a) Chemical structures of compounds used in this study. (b) Schematic illustration of the formation of DCPAs via ambient pressure drying. (c) Photograph showing DCPA-**3** resting on a gypsophila. (d) Photograph demonstrating the strength of DCPA-**3**, which is supporting over 3300 times its own weight. (e) Photographs of various shapes made from DCPA-**3**. (f) Processability of DCPA-**3** shown by cutting it into a butterfly with scissors. (g) Photograph of a piece of DCPA-**3** holding a 100 g load. (h) Photographs demonstrating the flexibility of DCPA-**3** by bending and knotting tests. (i) Photographs showing the rebound elasticity of DCPA-**3** in a free-falling process. The scale bars are 1 cm in (c) and (h), 5 cm in (e) and 4 cm in (f).

The chemical structures of the compounds used in this study and the fabrication process of DCPAs are illustrated in Fig. 1a and b. TA and DETA were firstly chosen to synthesize a polyimine oligomer (Fig. 1b). After prepolymerization, TREN was added as a cross linker (Fig. 1b). Due to the high reactivity between the aldehyde and amine groups, the sol–gel transition was observed ∼3 min prior to the macroscopic phase separation at 25°C (Fig. 1b). The aged gel (aging time = 48 h) experienced eight sequential solvent exchanges using a dimethylsulfoxide (DMSO)/anhydrous ethanol mixture (4 : 1, 1 : 1, 1 : 4 and 0:4, *v*/*v*) and an anhydrous ethanol/*n*-hexane mixture (4 : 1, 1 : 1, 1 : 4 and 0 : 4, *v*/*v*) within a period of 36 h at room temperature. Once the solvent exchanges and subsequent ambient pressure drying process (at room temperature for 12 h) were complete, DCPAs with a good appearance were obtained. On the one hand, the addition of TREN into the oligomer solution led to the formation of a 3D cross-linked network as a gel skeleton that could withstand capillary force during the drying process. On the other hand, the abundant imine bonds in the gel skeleton are able to undergo dynamic and reversible transimination reactions, thereby reinforcing the possibility of weldability, repairability and recyclability of the DCPAs. Under the constant 1 : 0.3 : 0.47 stoichiometry but variable concentrations of TA, DETA and TREN, we constructed monolithic DCPA-**1**, -**2** and -**3**, respectively (Table S1).

Taking DCPA-**3** as an example, in the Fourier-transform infrared (FT-IR) spectrum, the characteristic peak of the C=N stretch at 1640 cm^–1^ was prominent but the peak of the C=O stretch at 1690 cm^–1^ became very weak, suggestive of the formation of imine bonds (Fig. S1). The lightweight DCPA-**3** (119 mg/cm^3^) is able to rest on a fresh gypsophila bud (Fig. 1c). It can also support over 3300 times its own weight (300 mg, 17.8 mm (D) × 10.0 mm (H)) without obvious crack, demonstrating resistance capacity to compression (Fig. 1d). Due to the moderate reaction conditions of imine chemistry, great versatility in controlling the shapes of the DCPAs becomes possible. Monolithic DCPA-**3** samples of different shapes, such as a house, duck, horse and different letters, were readily prepared (Fig. 1e). Intriguingly, the desired shape, such as a butterfly, was readily made without any fractures by cutting with scissors (Fig. 1f), which reflects the processability of the DCPAs. Notably, most aerogels prepared by the sol–gel method are brittle and unmachinable [27–29]. A piece of DCPA-**3** (50 mg, 23 mm (L) × 7.0 mm (W) × 2.5 mm (H)) can sustain a load of 100 g without cracking, showing good tensile strength (Fig. 1g). In addition, DCPA-**3** displayed great flexibility (Fig. 1h). It not only could be folded in half, but it could also be knotted. Interestingly, DCPA-**3** can rebound without cracking after being released from a height of 25 cm and the rebound height was able to reach ∼9.0 cm (Fig. 1i). With these demonstrations, stereotypical characteristics regarding their lack of outstanding mechanical properties can be dismissed.

The basic properties of the DCPAs, including bulk density, porosity and shrinkage, are summarized in Table S1. The densities of the DCPAs ranged from 112.5 ± 2.6 to 121.4 ± 11.2 mg/cm^3^. The skeleton density of the polyimine was measured by a densimeter to be 1.30 g/cm^3^, resulting in high porosities of the DCPAs in the range of 90.7% to 91.3%. The shrinkage was evaluated by the linear shrinkage ratio (LSR). DCPA-**1** showed the most severe shrinkage (LSR = 22.8 ± 1.5%). This could be ascribed to the relatively weak gel skeleton caused by the low reactant concentration that failed to withstand the capillary force during the drying process. With the increase of the reactant concentration, the LSR of the DCPAs reduced accordingly. For example, DCPA-**3** showed the lowest LSR of ∼10.5 ± 2.8%, which was as good as those of aerogels (Fig. S2) prepared by supercritical drying and freeze-drying, in which the capillary force had been largely eliminated [30]. It is worth noting that no special treatments, such as hydrophobic modification, and no high-pressure or vacuum conditions were applied to the ambient-dried DCPAs to weaken the capillary force during the drying process [12]. Hence, the DCPAs have great potential to be fabricated on a large scale without any technological or device limitations.

### Morphologies and pore structures of the DCPAs

**Figure 2.**
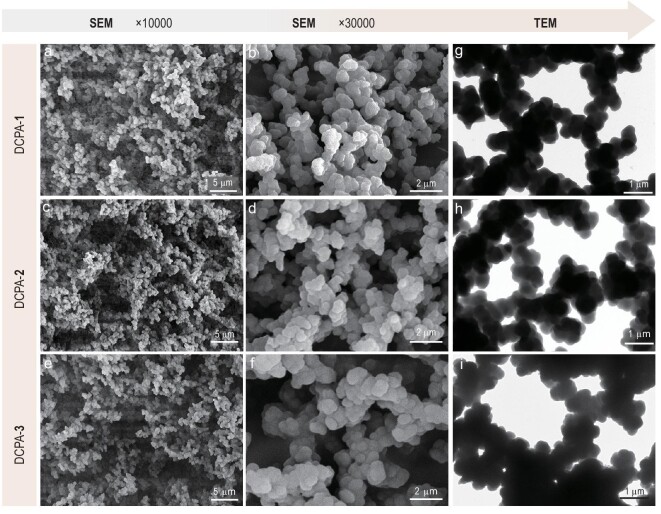
Structural characterization of the DCPAs. SEM images of (a and b) DCPA-**1**, (c and d) DCPA-**2** and (e and f) DCPA-**3**. TEM images of (g) DCPA-**1**, (h) DCPA-**2** and (i) DCPA-**3**.

The morphologies and specific surface areas of the DCPAs were investigated by scanning electron microscopy (SEM), transmission electron microscopy (TEM) and Brunauer-Emmett-Teller (BET) analysis, respectively. As shown in the SEM images, DCPA-**1** (Fig. 2a and b), DCPA-**2** (Fig. 2c and d) and DCPA-**3** (Fig. 2e and f) all displayed bi-continuous configurations consisting of polyimine gel skeletons and interconnected pores. On the one hand, the skeleton was constructed by interfused micro-nanostructured particles with well-dispersed sizes ranging from 400 to 800 nm (Fig. S3), indicative of typical pearl-necklace-like aerogel structures. On the other hand, the stacked micro-nanostructured particles formed the interconnected pores, ranging from hundreds of nanometers to a few microns (Fig. 2a–f). These results were further demonstrated by their TEM images (Fig. 2g–i). In the BET measurements, all of the nitrogen adsorption–desorption curves of the DCPAs showed a type III isotherm (Fig. S4). The specific surface areas (S_BET_) were measured to be 11.9 m^2^/g for DCPA-**1**, 11.7 m^2^/g for DCPA-**2** and 13.5 m^2^/g for DCPA-**3** (Table S1), which are similar to those of reported aerogels with similar morphologies [31].

### Fundamental mechanical properties of the DCPAs

Aerogels frequently show a trade-off between low density and high mechanical performance. To explore whether the DCPAs were capable of circumventing this inherent drawback, a series of compressive and three-point bending tests were performed. In sharp contrast to the brittle nature of most aerogels with morphological characters similar to the DCPAs [32,33]—for example, the mundane yet promising resorcinol–formaldehyde aerogels that opened the door to organic aerogels [27]—the DCPAs exhibited unexpected mechanical performance, including compressibility and flexibility.

**Figure 3.**
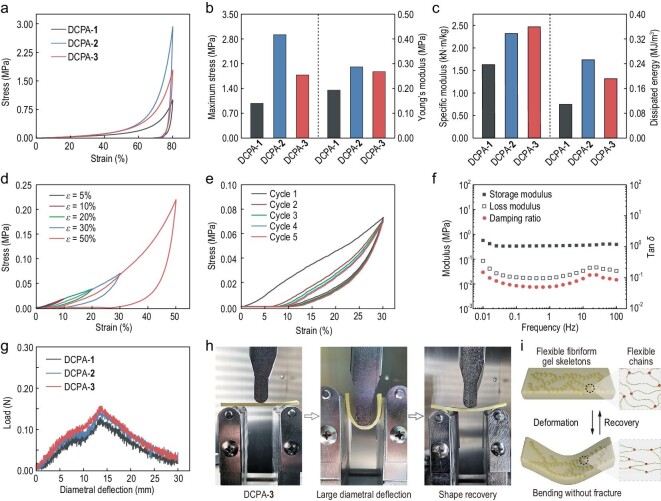
Mechanical properties of the DCPAs. (a) Compressive stress–strain curves of DCPAs at a strain of 80% with a deformation rate of 5.0 mm/min. (b) Maximum stress and Young's moduli of DCPAs. (c) Specific moduli and dissipated energy of DCPAs. (d) Compressive stress–strain curves of DCPA-**3** at strains of 5, 10, 20, 30 and 50%. (e) Cyclic compressive curves of DCPA-**3** at a strain of 30%. (f) Dynamic compressive behavior of DCPA-**3** with an oscillatory ɛ of 5% from 0.01 to 100 Hz. (g) Three-point bending curves of DCPAs and (h) corresponding bending images of DCPA-**3** with a deformation rate of 1.0 mm/min. (i) Schematic representation of the bending-induced deformation of the aerogel skeleton and corresponding polyimine networks.

The compressive stress–strain curves of the DCPAs at a strain of 80% are plotted in Fig. 3a. All of the DCPAs not only tolerated a high compressive strain without any cracks (Fig. S5), but also exhibited a nearly 10% recovery capacity after the pressure was released. Two characteristic deformation regimes, similar to other aerogels [34], were observed in the curves: an elastic regime with a linearly increased stress (<50% strain) and a densification regime with an exponentially increased stress (>50% strain).

The maximum stress and Young's moduli of DCPAs at a strain of 80% were obtained via their compressive curves (Fig. 3b). DCPA-**2** exhibited the greatest maximum stress of 2.92 MPa; the values of DCPA-**1** and DCPA-**3** were 0.98 and 1.80 MPa, respectively. This may be ascribed to the varying densities of these DCPAs (Table S1). Nevertheless, the Young's moduli of DCPA-**2** (0.287 MPa) and DCPA-**3** (0.267 MPa) are quite similar, which may be due to their similar gel skeletons. Subsequently, the specific modulus, defined as the ratio of the Young's modulus to the density, was calculated (Fig. 3c), and represents a significant parameter for light materials. The specific moduli of DCPA-**2** and DCPA-**3** were 2.30 and 2.45 kN · m/kg, respectively, demonstrating that the DCPAs possess a good anti-compression capacity (Figs 1d and S6). Moreover, we calculated the energy dissipation of the DCAPs based on the area of the hysteresis loop and the values were 0.11 MJ/m^3^ for DCPA-**1**, 0.25 MJ/m^3^ for DCPA-**2** and 0.19 MJ/m^3^ for DCPA-**3** (Fig. 3c). This indicates that the DCPAs are promising candidates for cushioning materials [10]. We also fabricated the aerogel under highly humid conditions (RH 95%) and the resultant aerogel was denoted as DCPA-**2** (RH 95%) (Fig. S7). With the exception of the lowered maximum stress derived from the influence of the high humidity on the imine bonds, DCPA-**2** (RH 95%) was the same as the original DCPA-**2** in terms of its appearance and microstructure. In consideration of DCPA-**3** with more balanced properties, the following tests focused on further investigation of the mechanical properties of the DCPAs.

The compression tests of DCPA-**3** at different strains of 5, 10, 20, 30 and 50% were carried out (Fig. 3d). When the strain was <20%, the curves almost overlapped, indicating that DCPA-**3** had decent elasticity in a certain range (Fig. 3d). Upon increasing the strain to 50%, DCPA-**3** still showed considerable recovery capacity. Similar compressive stress–strain curves were also observed for DCPA-**1** and DCPA-**2** (Fig. S8). Furthermore, the compression fatigue test of DCPA-**3** was carried out with five loading–unloading cycles at a 30% strain without an interval (Fig. 3e). DCPA-**3** could retain 95% of the original maximum stress and the maximum volume deformation was 9.2% (Figs 3e and S9), suggestive of a certain degree of fatigue resistance. A dynamic compressive test was further performed on DCPA-**3** by dynamic mechanical analysis (DMA). The storage modulus remained almost stable over four orders of magnitude from 0.01 to 100 Hz, and the loss modulus varied by only one order of magnitude (Fig. 3f), indicating that an elastic response existed in the DCPAs.

The three-point bending curves of diametral deflection as a function of load force were measured to showcase the flexibility of the DCPAs (Fig. 3g). After a large diametral deflection of 30 mm with a fixture span of 25 mm, all of the DCPAs nearly recovered to their original states without any cracks (Figs 3h and S10). The bending flexibility of the DCPAs reached the level of the reported flexible aerogels [35]. Notably, flexibility in compression and bending with large strains has rarely been realized in aerogels prepared by the sol–gel method [35]. The mechanical performance of the DCPAs may originate from two key aspects (Fig. 3i): (i) unlike the traditional silica aerogels with rigid Si–O–Si networks, and classic resorcinol–formaldehyde aerogels with short aliphatic hydrocarbon chains [36,37], the DCPAs contain relatively flexible chains in the networks [35]; (ii) the interfused micro-nanostructured polyimine particles are able to evolve into fibriform gel skeletons that can promote recovery after compression and bending tests.

### Weldability, repairability and recyclability of the DCPAs

**Figure 4.**
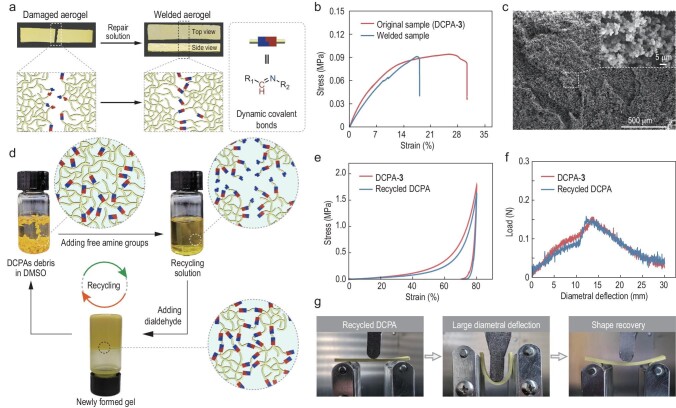
Weldability and recyclability of the DCPAs. (a) Photographs and schematic representation of the welding process of DCPA-**3**. (b) Tensile stress–strain curves of the virgin and welded samples with a deformation rate of 5.0 mm/min. (c) SEM images of the fracture surface of the welded sample. (d) Schematic representation of the recycling process of DCPA-**3**. (e) Compressive stress–strain curves of DCPA-**3** and the recycled DCPA at a strain of 80% with a deformation rate of 5.0 mm/min. (f) Three-point bending curves of DCPA-**3** and the recycled DCPA with a deformation rate of 1.0 mm/min, and (g) corresponding bending photographs.

Considering the abundant dynamic bonds in the DCPAs, it was expected that the DCPAs would exhibit emerging features that are rarely possessed by traditional aerogels. As a first trial, the weldability of the DCPAs was tested. By applying a small amount of fresh polyimine sol at the cut DCPA-**3**, followed by solvent exchange and ambient pressure drying, the cut aerogel chips were welded (Fig. 4a). In the welded area, the fracture surface was barely observed from both the top and side views (Fig. 4a). To prove this effect, the tensile behaviors of DCPA-**3** before and after welding were studied (Fig. 4b). Compared with the original tensile curve of DCPA-**3**, the welded specimen showed good recovery efficiencies in terms of different parameters, including fracture stress (97%) (Fig. S11), strain at break (60%) (Fig. S12) and elastic modulus (80%) (Fig. S13). Furthermore, the SEM image showed that the welded surface formed similar morphologies to those observed in DCPA-**3** (Fig. 4c), thus facilitating the fusion between the fragmented aerogels [19]. This can be explained by the fresh polyimine growing across the fracture sites of the DCPA-**3** via dynamic imine bonds so as to promote the welding effect [38]. Furthermore, we demonstrated the repairability of the DCPA-**3** (Fig. S14). After being repaired using polyimine solution, the scratches on the surface of the aerogel film disappeared (Fig. S14a). Even in the SEM image, no obvious cracks or scratches were observed (Fig. S14b).

Subsequently, we explored the recyclability of the DCPA-**3** by adding an excess of free amine groups to disrupt the stoichiometric balance between the aldehyde and the amine groups and induce transimination reactions, which could decrease the molecular weight and solubilize the polymer network. Specifically, a certain amount of diamine and/or triamine monomers in DMSO as the recycling solution (see Supplementary Data for details) was mixed with the debris of DCPA-**3** (Fig. 4d). After heating and ultrasonic treatments, the DCPA degraded into soluble oligomers/monomers, which could regenerate the polyimine gel upon the addition of the dialdehyde monomer in proportion (Fig. 4d). Subsequently, the recycled DCPA was obtained via ambient pressure drying. The LSR of the recycled DCPA was only 15.9 ± 1.4% (Table S1), representing a satisfactory result. The morphology of the recycled DCPA retained the same pearl-necklace-like structure formed by the interfused micro-nanostructured particles (Figs S3 and S15). The mechanical properties of the recycled DCPA were also explored. The compressive strain–stress curve of the recycled DCPA almost overlapped with that of the original DCPA-**3** (Fig. 4e). Compared with DCPA-**3**, the maximum stress (Fig. S16) and compression moduli (Fig. S17) of the recycled DCPA recovered to 92% and 73%, respectively. The three-point bending curve further proved that the recycled DCPA maintained desirable mechanical performance (Fig. 4f and g). The emerging features of weldability, repairability and recyclability have potential value with regard to sustainable development, and add a new research perspective for both aerogels and dynamic covalent polymers.

### Multifunctionality of the DCPAs

In the preceding sections, we focused on the basic properties, mechanical properties and emerging features, including weldability, repairability and closed-loop recyclability, of the DCPAs. In this section, we will explore the multifunctionality of the DCPAs, including thermal insulation and oil–water separation, by taking advantage of their inherent porous structures and robust skeletons.

**Figure 5.**
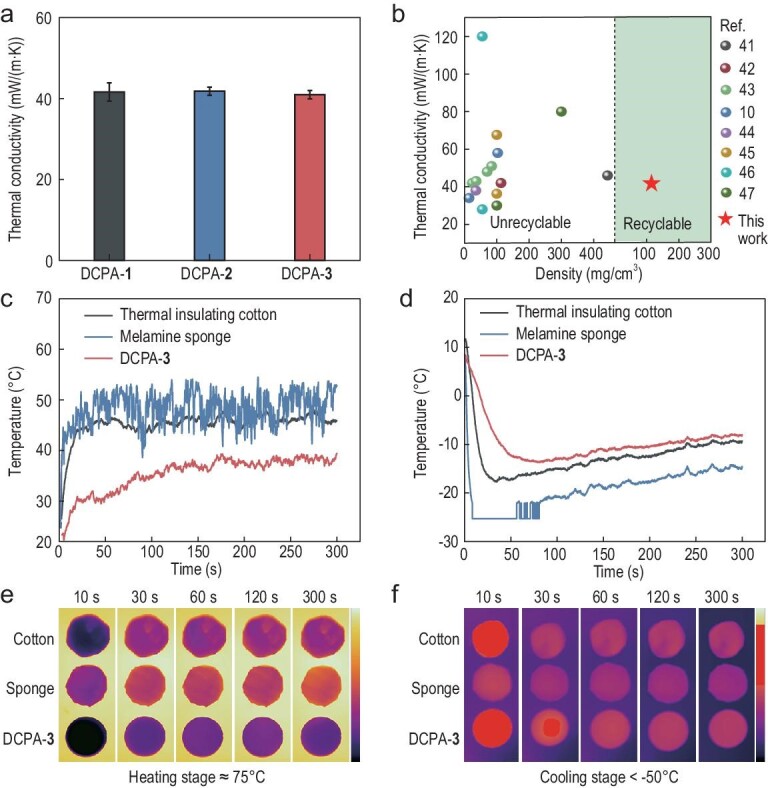
Thermal insulation of the DCPAs. (a) Thermal conductivities of DCPAs measured at room temperature by the transient plane source method. (b) Ashby plot of the thermal conductivity and density of DCPAs and other reported aerogels, including PMSQ aerogel [41], PVA aerogel [42], PU/alumina aerogel [43], MXene/PI aerogel [10], 3D ordered nanofiber aerogel [44], polymeric woods [45], CNF aerogel [46] and polyamide aerogel [47]. (c) Temperature variation curves of the back side of the commercial thermal insulting cotton, melamine sponge with high porosity, and DCPA-**3** on a 75ºC stage for 300 s. (d) Temperature variation curves of the back side of commercial thermal insulting cotton, melamine sponge with high porosity, and DCPA-**3** on a cold stage (<−50ºC) for 300 s. Corresponding infrared images of the three samples on (e) a 75ºC stage and (f) a cold stage (<−50ºC).

First of all, we measured the thermal conductivities of DCPA-**1**, DCPA-**2** and DCPA-**3** as 41.6 ± 2.3, 41.8 ± 1.0 and 40.9 ± 1.0 mW/(m · K), respectively (Fig. 5a). These values are comparable or even superior to those of most aerogels (Fig. 5b). The total thermal conductivity (λ_total_) of the aerogel is the arithmetic sum of the radiative heat transfer coefficient (λ_r_), solid thermal conductivity (λ_s_) and gas thermal conductivity (λ_g_), which reflects the real thermal transfer coefficient [39,40]. The low densities lead to a low λ_s_ and the small pore size results in the decrease of λ_g_, which jointly accounted for the low thermal conductivities of the DCPAs (see Supplementary Data for a detailed discussion).

Given the good performance of thermal conductivity, we proceeded to probe the real thermal insulation of DCPA-**3** with an infrared camera and compared it with other common thermal insulating materials, including commercial cotton and melamine sponge, with the same thickness (5.0 mm). The measured samples were placed on a 75ºC heating stage. As shown in the time–temperature curves and the thermal infrared images, the far-end surface temperatures of the commercial thermal insulating cotton and melamine sponge rose quickly within 25 s and the thermal equilibrium time was <50 s (Fig. 5c and e). In contrast, the far-end surface temperature of DCPA-**3** increased more slowly and the thermal equilibrium time extended to ∼144 s with a lower equilibrium temperature (Fig. 5d). Furthermore, the samples were placed on a cold stage (<50ºC) to study the thermal insulation in a low-temperature environment (Fig. 5d and f). The far-end surface temperature of the melamine sponge decreased rapidly to −25ºC within 8 s. For DCPA-**3**, it took a longer time (85 s) to reach the lowest temperature of only −13.5ºC. The thermal insulation performance of the DCPAs at both high and low temperatures was consistent with their low thermal conductivities.

**Figure 6.**
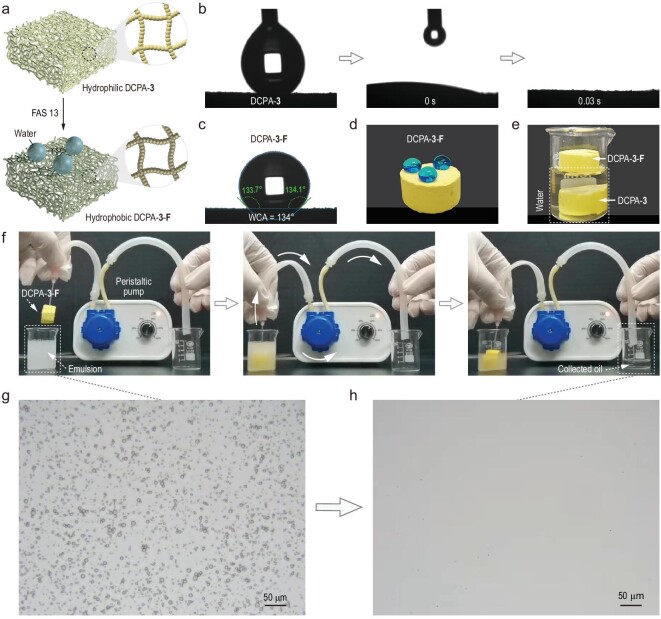
DCPAs for emulsion separation. (a) Schematic representation of the hydrophobic modification process of DCPA-**3**. The water contact angle (WCA) measurements of (b) DCPA-**3** and (c) DCPA-**3**-**F**. Photographs demonstrating (d) the hydrophobicity of DCPA-**3**-**F** and (e) the hydrophilicity of DCPA-**3**. (f) The continuous oil–water emulsion separation apparatus driven by a peristaltic pump. Corresponding optical images (g) before and (h) after separation.

As they benefited from plenty of micro-nanostructured particles in the aerogel frameworks, we conjectured that the DCPAs could be modified readily into hydrophobic materials by low surface energy chemicals [48]. The modification of DCPA-**3** was achieved by introducing the hydrophobic surface via the condensation of fluoroalkyl silane (FAS 13) on the aerogel skeleton, which was denoted as DCPA-**3**-**F** (Fig. 6a). The energy dispersive spectroscopy (EDS) mapping images clearly show that the Si and F elements were well dispersed in the aerogel matrix (Fig. S18). Before hydrophobic modification, the water droplet was adsorbed rapidly by the hydrophilic DCPA-**3** during the water contact angle (WCA) measurement (Fig. 6b). In sharp contrast, the WCA of DCPA-**3**-**F** could reach up to 134º (Fig. 6c). More intuitively, the near-spherical water droplets (0.3 mL/droplet) were capable of being supported by DCPA-**3**-**F** (Fig. 6d) and DCPA-**3**-**F** could keep afloat in the water (Fig. 6e), exhibiting the hydrophobicity of the modified DCPA. In light of its high porosity and lipophilicity, DCPA-**3**-**F** was then exploited to separate the immiscible oil–water mixtures. As shown in Fig. S19, DCPA-**3**-**F** could rapidly remove both the light oil on top of the water (*n-*hexane dyed with oil red) and the heavy oil under the water (dichloromethane dyed with oil red). The success led us to attempt the separation of a surfactant-stabilized emulsion, which is more valuable and challenging for aerogels. As such, DCPA-**3**-**F** was equipped with a peristaltic pump to construct a simple and continuous separation device by the pumping method (Fig. 6f). When DCPA-**3**-**F** was added to the surfactant-stabilized W/O emulsion (water/*n*-hexane), the turbid emulsion was purified into a clear oil (Fig. 6f and Movie S1), which was further confirmed by the corresponding optical images (Fig. 6g and h). Moreover, the surfactant-stabilized water/toluene and water/petroleum ether emulsions were also successfully separated by DCPA-**3**-**F** (Fig. S20 and Movie S1). Such a simple and continuous pumping method for emulsion separation implies that the DCPAs have potential application in pollution control and environmental protection fields [49].

## CONCLUSION

In summary, we demonstrated that DCPNs, an increasingly important research topic, can be employed to fabricate monolithic DCPAs with newly developed features, such as weldability, repairability, degradability and closed-loop recyclability. The mild and catalyst-free sol–gel process via dynamic imine chemistry and the simple ambient pressure drying method make it possible to fabricate DCPAs in large quantities with arbitrary models. Furthermore, the DCPAs not only possessed a low density of ∼119 mg/cm^3^ and a suppressed linear shrinkage rate of ∼10.5%, but also showcased a basketball-like rebound ability, large degrees of compression (80% strain) and bending (diametral deflection of 30 mm) without any cracks, and a decent tensile strain of 32.7%. In addition, on account of their closed-loop chemical recyclability and robust porous structures, the DCPAs can be developed into green, low-cost and multifunctional materials for thermal insulation and water treatment. This straightforward principle would also be suitable for other dynamic covalent bonds, to enrich the library of DCPAs. We expect that this work will facilitate the development of both dynamic covalent chemistry and aerogels for applications as smart and environmentally friendly materials.

## Supplementary Material

nwac012_Supplemental_Files
