# Supplementary material for: Weldable and closed-loop recyclable monolithic dynamic covalent polymer aerogels
Source: Natl Sci Rev. 2022 Jan 28;9(9):nwac012. doi: 10.1093/nsr/nwac012 (PMC9564194; doi:10.1093/nsr/nwac012)
Supplement: nwac012_Supplemental_Files [file nwac012_supplemental_files.zip › NSR_MS-2021-1110.R1_Supporting_Information.docx]

Supporting Information

Weldable and closed-loop recyclable monolithic dynamic covalent polymer aerogels

Xinhai Zhang^1^, Jun Zhao^1^, Kai Liu^1^, Guangfeng Li^1^, Dong Zhao^1^, Zhaoming Zhang^1^, Junjun Wan^1^, Xue Yang^1^, Ruixue Bai^1^, Yongming Wang^1^, Wei Zhang^2,^* and Xuzhou Yan^1^^,^*

*^1^School of Chemistry and Chemical Engineering, Frontiers Science Center for Transformative Molecules, Shanghai Key Laboratory of Electrical Insulation and Thermal Aging, Shanghai Jiao Tong University, Shanghai 200240, P. R. China*

*^2^Department of Chemistry**, University of Colorado Boulder, Boulder, CO 80309, USA*

*Correspondence authors. E-mails: xzyan@sjtu.edu.cn, wei.zhang@colorado.edu

1. Experimental Procedures

1.1 Materials

Terephthalaldehyde (TA, 97%), diethylenetriamine (DETA, 99%), and tris(2-aminoethyl)amine (TREN, 98%) were purchased from TCI (Shanghai) Development Co., Ltd. Dimethyl sulfoxide (DMSO, 99.7%), anhydrous ethanol (99.7%), n-hexane (97.5%), toluene (98.5%), petroleum ether (98.5%), and dichloromethane (DCM, 99.5%) were purchased from Sinopharm Chemical Reagent Co., Ltd. 1H,1H,2H,2H-perfluorooctyltrichlorosilane (FAS 13) was purchased from Zhejiang Research Institute of Chemical Industry Co., Ltd. The involved water in this work was deionized water. All of the chemicals were commercially available and they were used as supplied without further purification.

1.2 Fabrication of dynamic covalent polymer aerogels (DCPAs)

Typically, TA (0.50 g, 3.73 mmol) and DETA (0.12 g, 1.12 mmol) were mixed in a glass bottle with 6.8 mL of DMSO at room temperature. After stirring for 6 h, polyimine oligomer sol was obtained. Then, TREN (0.25 g, 1.74 mmol) was added to the sol to result in the gelation at 25 °C. After aging for 48 h, the resultant gel experienced eight sequential solvent exchanges by a DMSO/anhydrous ethanol mixture (4:1, 1:1, 1:4, and 0:4, *v*/*v*) and an anhydrous ethanol/*n*-hexane mixture (4:1, 1:1, 1:4, and 0:4, *v*/*v*) within a period of 36 h at room temperature and then dried for 12 h at ambient pressure and room temperature. A series of dynamic covalent polymer aerogels (DCPAs) were prepared using the same procedure and denoted as DCPA-**1**, DCPA-**2**, and DCPA-**3**, respectively. The detailed information was summarized in Table S1.

1.3 Weldability, repairability, and recyclability experiments

For the weldability test, two pieces of the cut DCPA-3 were placed closely and then dropped the mixture solution of polyimine oligomer sol and TREN. A certain of force was applied on the samples to ensure good contact. Then the welded DCPA-**3** was placed at ambient environment for aging and multistep solvent exchange as described above. The repairability experiment is similar to that of weldability process.

For recyclability experiment, the recycling solution was composed of 47.0 mg of DETA, 104 mg of TREN, and 3.74 mL of DMSO. Then, DCPA-3 (93.0 mg) was added in the recycling solution. The mixture was stirred under heating and ultrasonic conditions to depolymerize the polymer networks of the DCPA into soluble monomers/oligomers. TA (200 mg) dissolved in 0.4 mL of DMSO was added into the mixture to consume the free amine groups and then resulted in the formation of the recycled DCPA after solvent exchange and ambient pressure drying.

1.4 Hydrophobic modification of DCPA-3

FAS 13 (500 mg) was mixed with 100 mL of the mixture of anhydrous ethanol and water (99:1 v/v) for hydrolyzation at 50 °C for 2 h. Then the hydrolysate of FAS 13 was added into the DMSO in batches in the preliminary solvent exchange process to modify the DCPA-**3**. The modified DCPA-**3** was denoted as the DCPA-**3**-**F**.

The n-hexane, toluene, petroleum ether, and water were used to prepare W/O emulsions by mixing emulsifier (Span 80, 1.0 mg/mL). The volume ratio of the oil phase and water was 99:1. Typically, the Span 80 and water was added dropwise into the n-hexane under vigorous stirring conditions. Then ultrasonic treatment (2 h) was applied to form a stable milky emulsion.

1.5 Characterization

Fourier Transform Infrared Spectroscopy (FT-IR) measurements were conducted using a Nicolet 6700 spectrometer (Thermo Fisher, USA), and the wavenumber ranges from 500 to 4000 cm^-1^ with 32 scans at a resolution of 4.0 cm^-1^. The N_2_ adsorption/desorption isotherms of the DCPAs were obtained by a N_2_ adsorption analyzer (BELSORP-MAX, Japan) and the aerogel sample was degassed at 100 °C for 12 h before measurement. The skeleton density was measured by a densimeter (XF120S, China). Morphological structures of the prepared samples were observed by field emission scanning electron microscope (FESEM, Nova NanoSEM 450, USA) and transmission electron microscopy (TEM, Tecnai G2 SpiritBiotwin, USA). The compression tests, extension tests, and three-point bending tests were performed on a universal testing machine with a micro-computer control system (WDW-5, Shanghai Songdun). The running speed was 5.0 mm/min for compression and extension tests. The running speed was 1.0 mm/min for three-point bending tests and the size of the tested aerogels is 60 (L) × 10 (W) × 2.0 mm (H). The apparatus span was set as 25 mm and the diametral deflection was 30 mm. The dynamic compressive behavior was measured by a dynamic mechanical analyzer (DMA, Discovery DMA 850, USA) with a prestrain of 5% and 20 μm amplitude. The thermal conductivities of the aerogels were measured at ambient conditions using a hot disk thermal analyzer (TPS 2500S, Sweden) that is according to ISO 22007-2. An infrared camera (FORTIC 340, Shanghai FORTIC INC., China) with a micro-computer control system was selected to record the thermographic images and the real-time temperatures of the aerogels placed on a hot or cold stage. The cold stage was taken out of liquid nitrogen after cooling for 3 mins. The emulsion droplets were observed using optical microscope (Hirox KH7700, USA). The hydrophobicity of the aerogels was evaluated based on water contact angles (WCA) of the aerogels that were measured using a contact angle analyzer (DSA100, Germany).

1. Results and Discussion


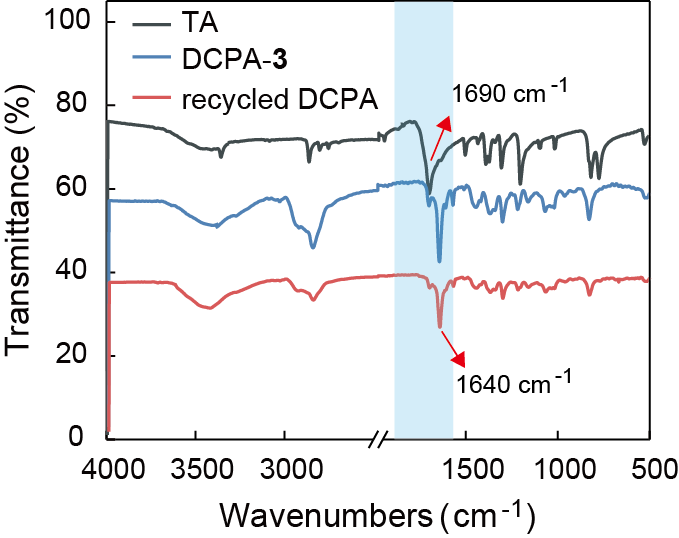


**Figure S1.** The FT-IR spectra of TA, DCPA**-3**, and recycled DCPA.


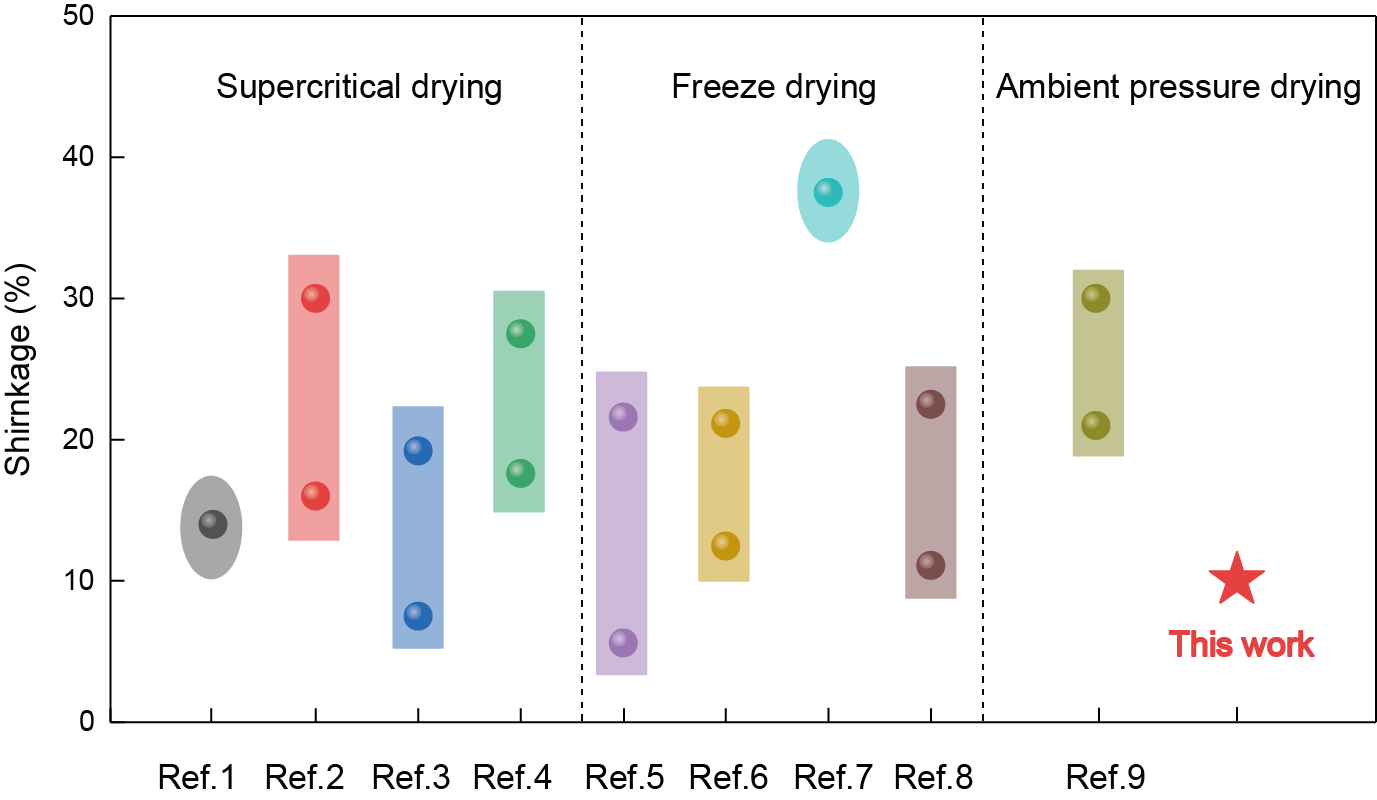


**Figure S2.** Comparison of the linear shrinkage of DCPA-**3** with other aerogels, including LC-NCell aerogel [1], polyimide/ZIF composite aerogels [2], Polyimide-based aerogels [3], Polyamide aerogels [4], 3D-printed cellulose nanofibril monolith [5], 3D-printed CNC aerogels [6], Polyimide/CNT Composite aerogel [7], ANF/polyimide aerogels [8], PVPMS aerogels [9].


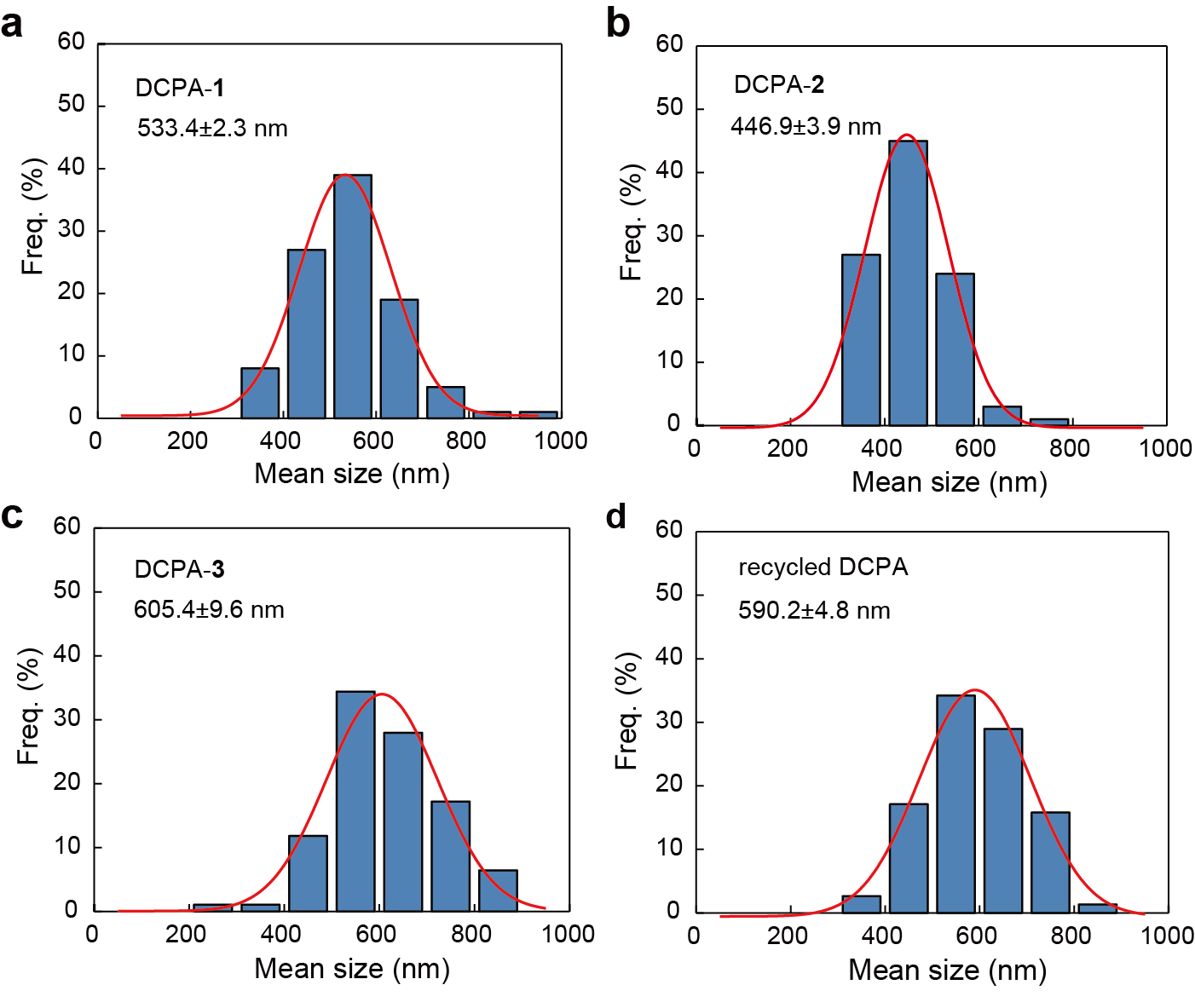


**Figure S3.** Particle size distributions of DCPAs and recycled DCPA, acquired by a Nano Measurer1.2.


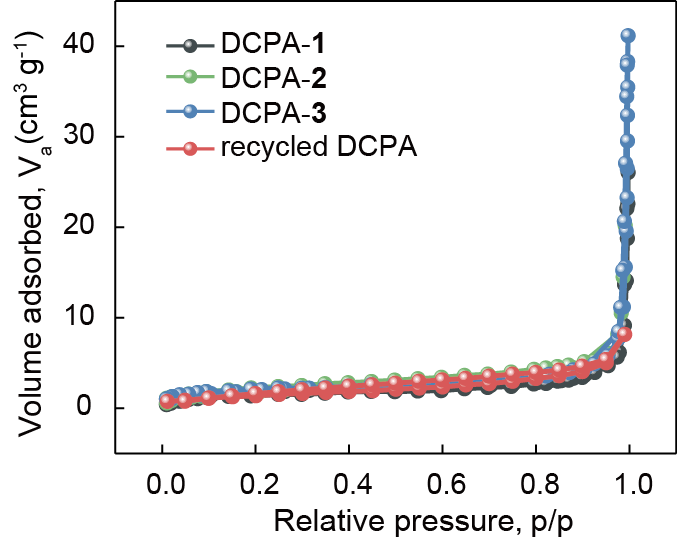


**Figure S4.** Nitrogen adsorption-desorption isotherms of DCPAs and recycled DCPA.


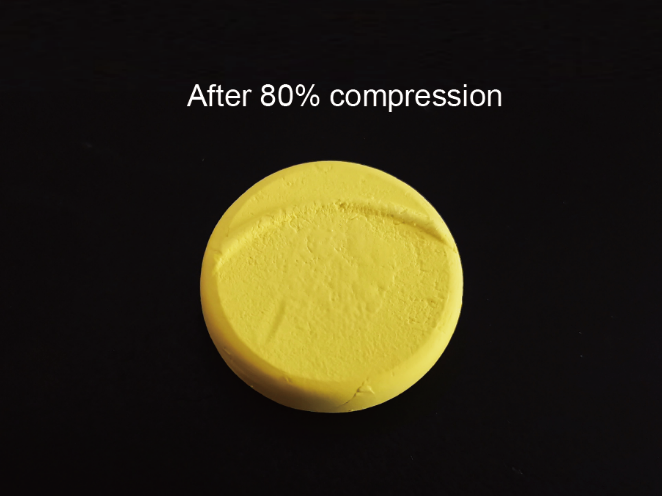


**Figure S5.** Photograph of appearance of the DCPAs after 80% compression.


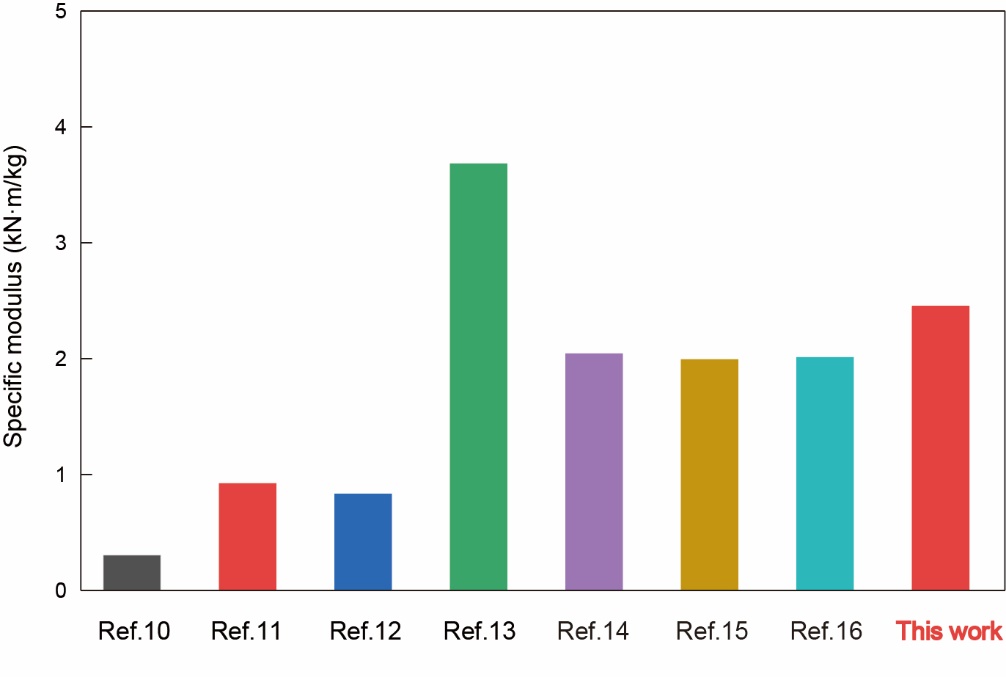


**Figure S6.** The specific modulus comparison of the DCPA-3 with the reported polymer-based aerogels with considerable flexibility, such as GO/polyimide aerogel [10], SiO_2_/RF aerogel [11], BN/polyimide aerogel [12], RF aerogel [13], Aramid aerogel [14], Aramid nanofibers/Ti_3_AlC_2_ [15], and BNAF aerogel [16].

**
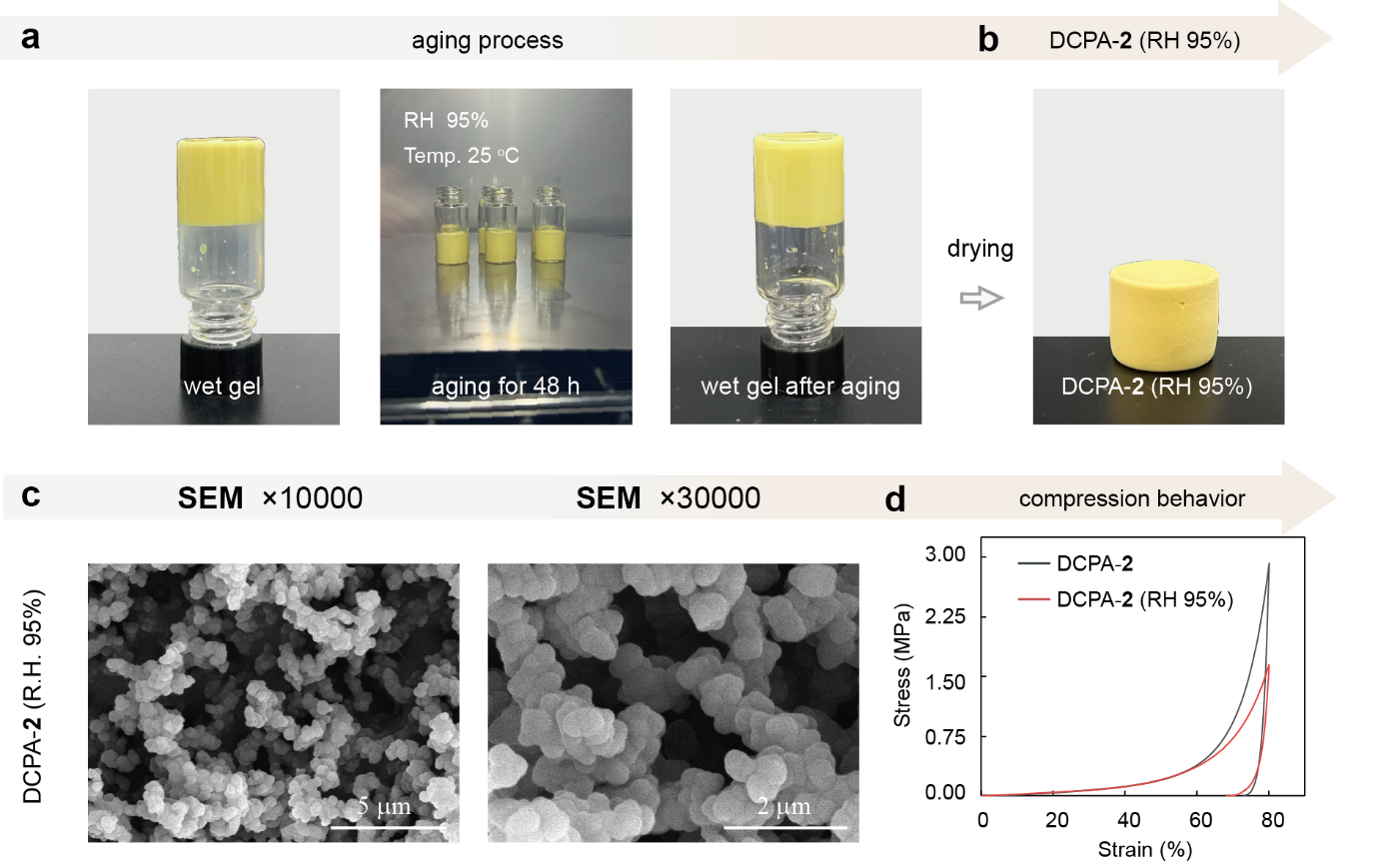
**

**Figure S7.** (a) Photographs of the aging process of the polyimine wet gel at a high relative humidity of 95% at 25 °C. (b) Photograph of the corresponding aerogel (DCPA-**2** (RH 95%)). (c) SEM images of DCPA-**2** (RH 95%). (d) Compressive stress–strain curves of DCPA-**2** and DCPA-**2** (RH 95%) at a strain of 80% with a deformation rate of 5.0 mm/min. According to the gel images, the polyimine wet gel still showed considerable integrality after aging at a high relative humidity of 95% at 25 °C for 48 h. The resultant DCPA-2 (RH 95%) aerogel also showed good appearance and was same as the original DCPA-**2** in terms of its appearance and microstructure. However, the maximum stress is lower than that of DCPA-**2**. It may be due to the influence of high humidity on the imine bonds.


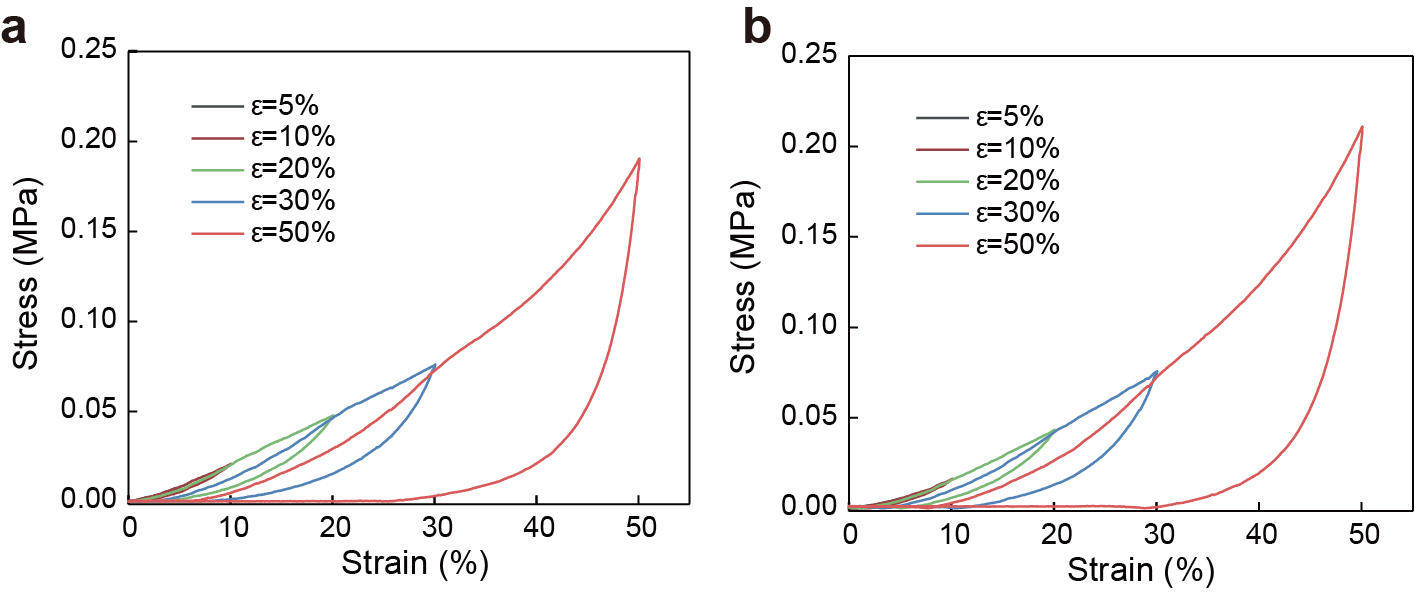


**Figure S8.** Compressive stress–strain curves of (a) DCPA-**1** and (b) DCPA**-2** under different strains. Deformation rate: 5.0 mm/min.


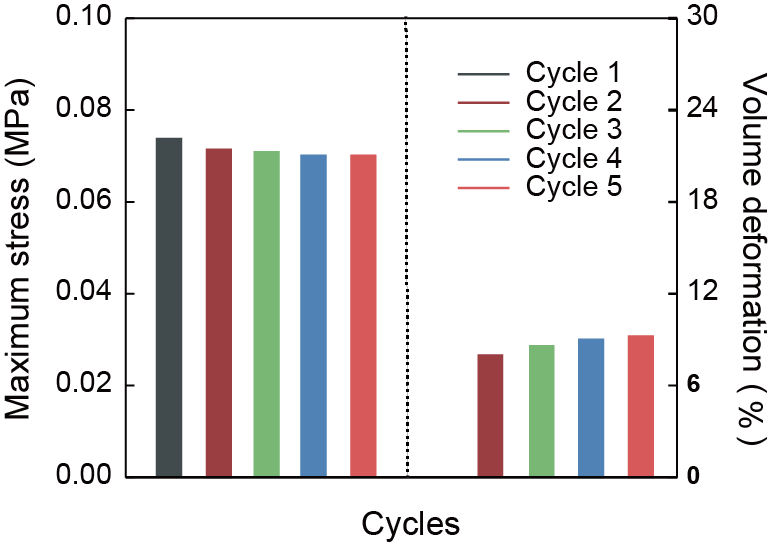


**Figure S9.** Variations of maximum stress and volume deformation of DCPA-**3** in cyclic compression under a strain of 30%.


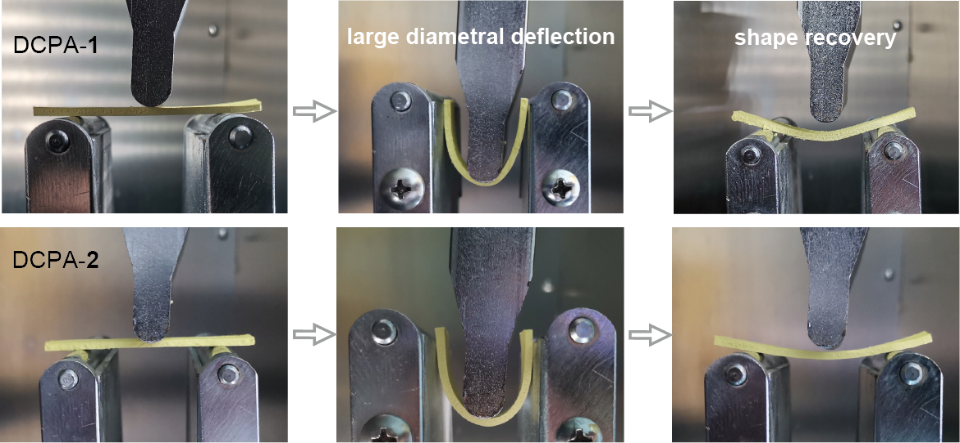


**Figure S10.** Digital images of three-point bending tests of DCPA**-1** (top) and DCPA**-2** (down).


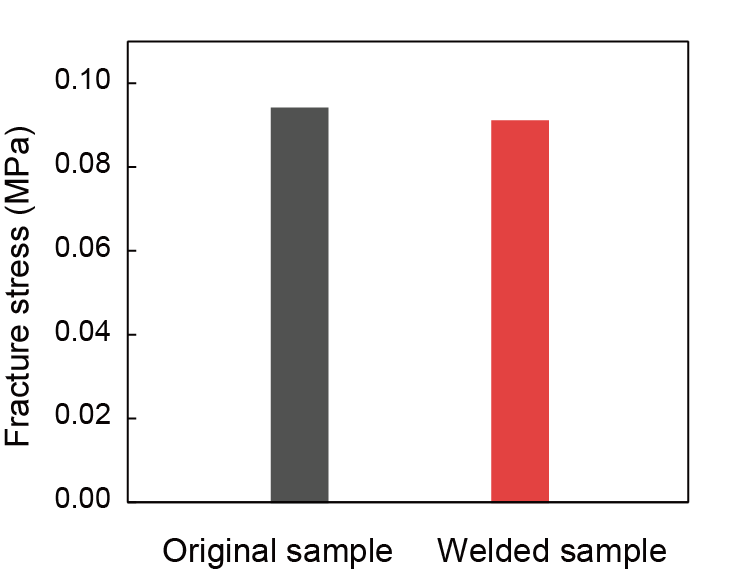


**Figure S11.** Fracture stress of the virgin and welded samples.


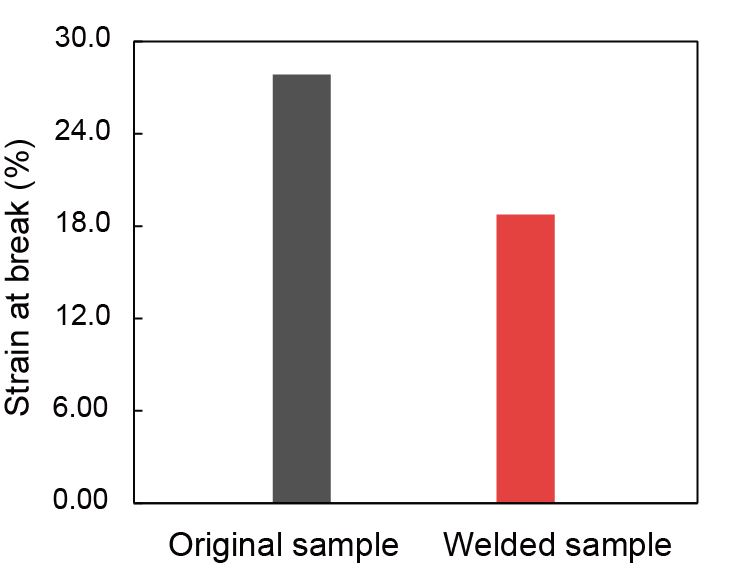


**Figure S12.** Strain at break of the virgin and welded samples.


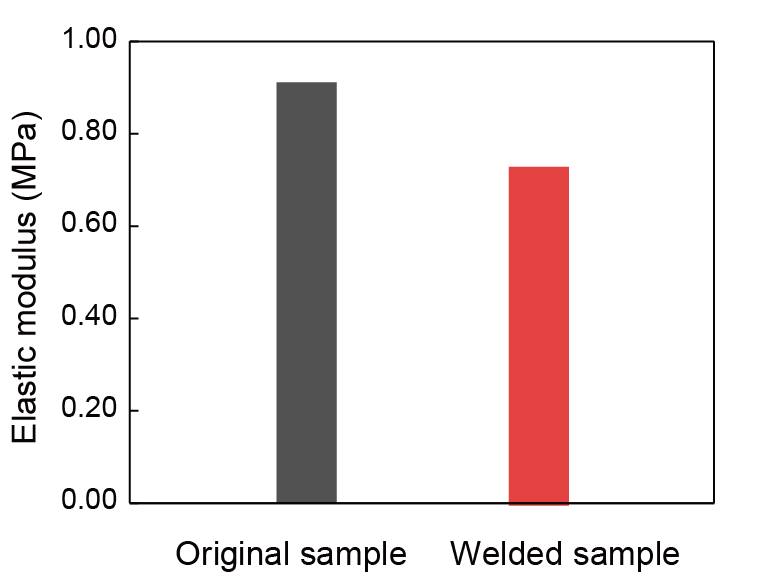


**Figure S13.** Elastic modulus of the virgin and welded samples.


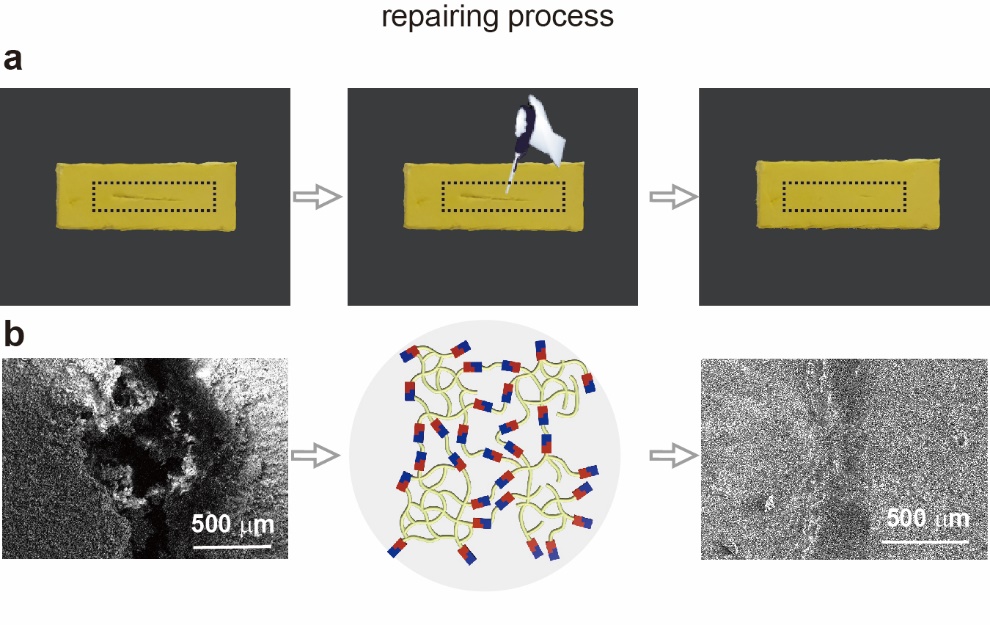


**Figure S14.** (a) Schematic representation of the repairing process of DCPA-**3** and (b) SEM images of the repaired DCPA.


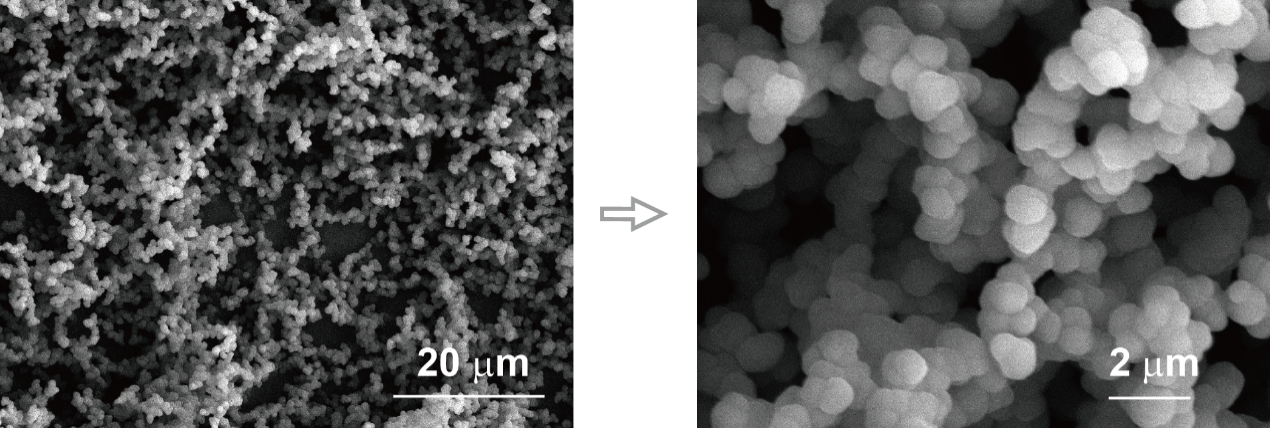


**Figure S15.** SEM images of the recycled DCPA.


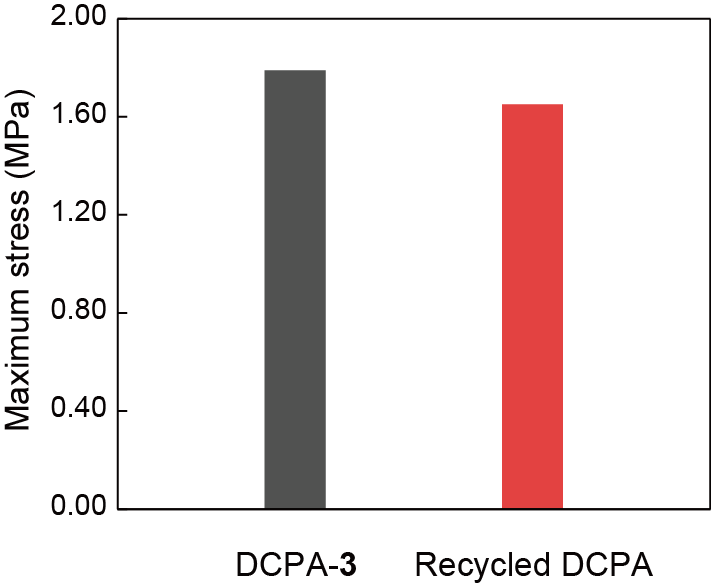


**Figure S16.** Maximum stress of DCPA**-3** and the recycled DCPA at a strain of 80%.


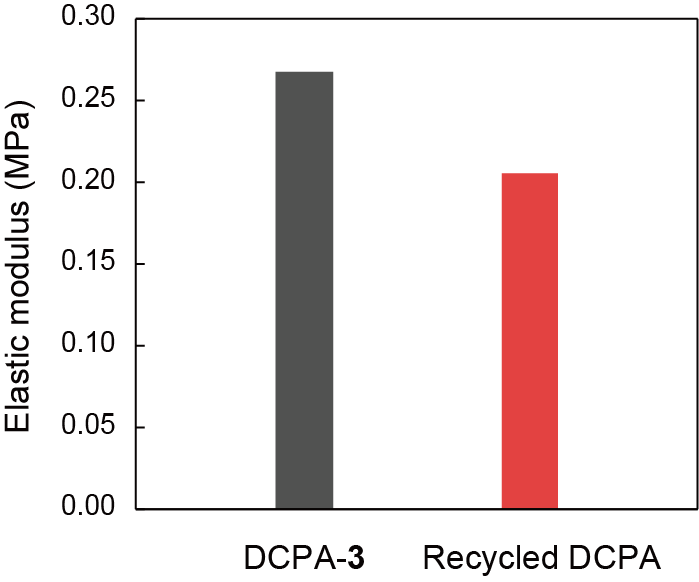


**Figure S17.** Elastic modulus of DCPA-**3** and the recycled DCPA.


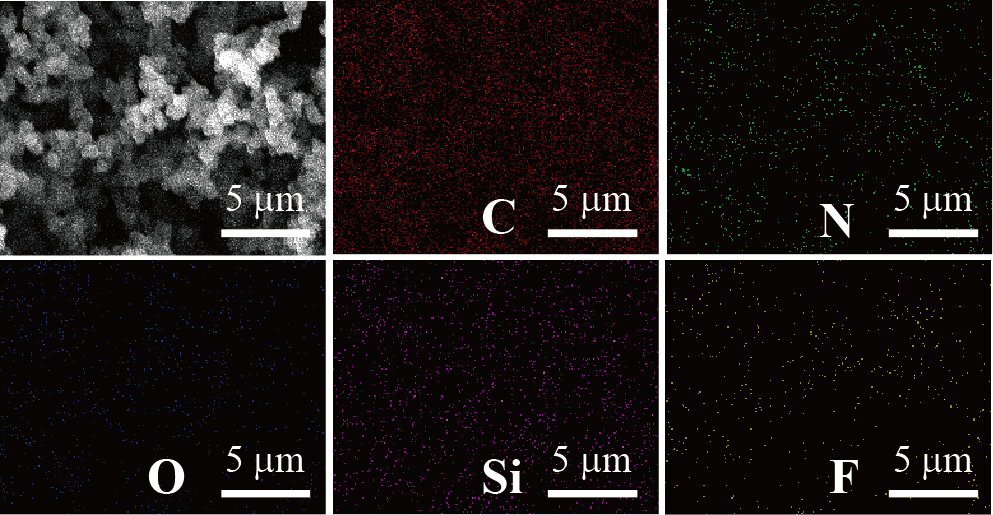


**Figure S18.** Typical SEM and elemental mapping of the DCPA-**3**-**F** confirming the existence of the F and Si elements that are characteristic elements of FAS 13.


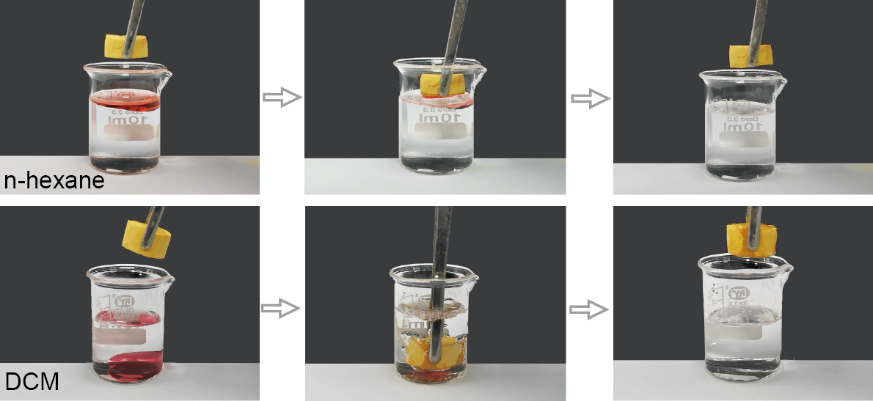


**Figure S19.** Digital images of selective absorption of light oil (*n*-hexane) atop the water and heavy oil (DCM) under the water.


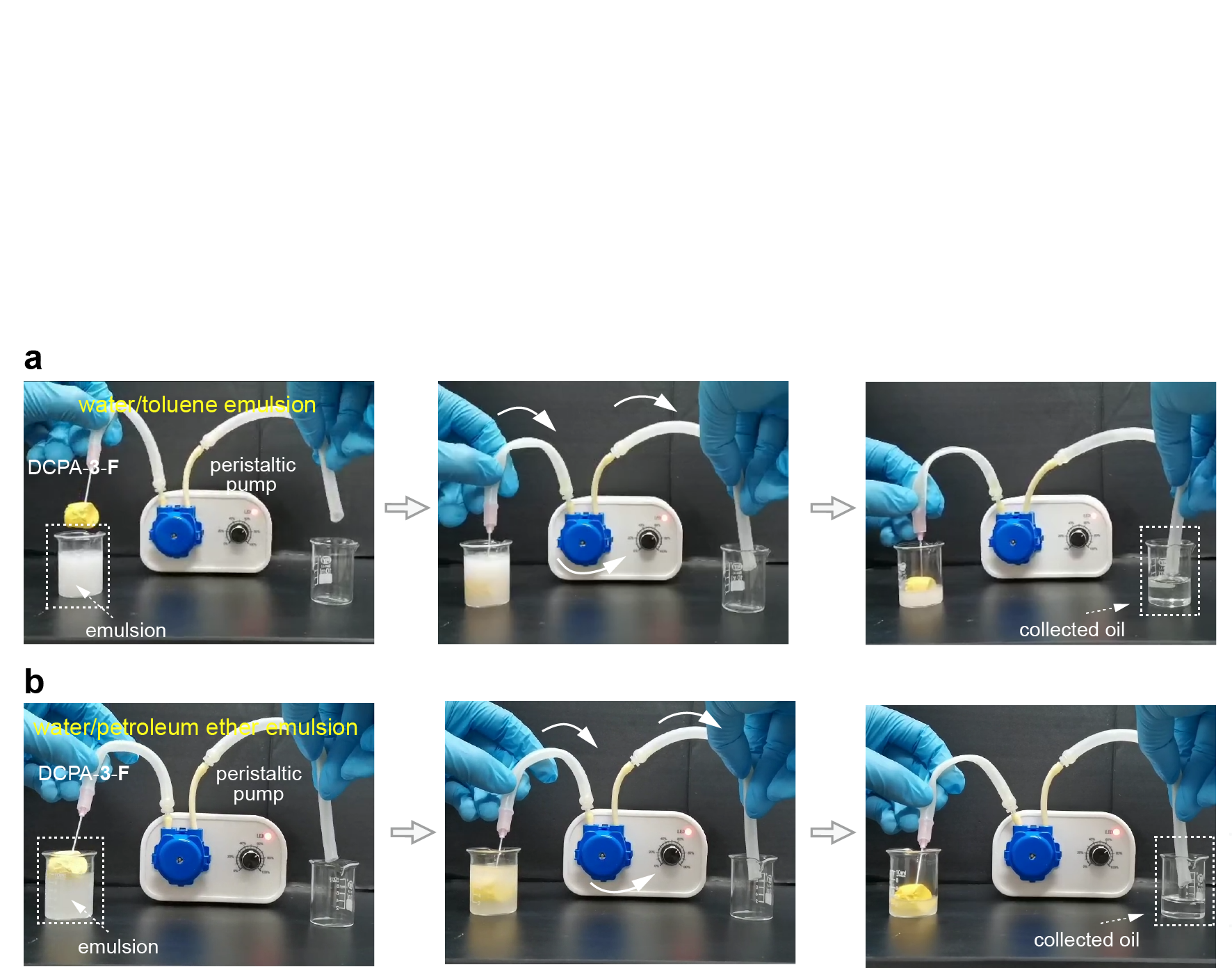


**Figure S20.** The continuous oil-water emulsion separation apparatus driven by a peristaltic pump. (A) water/toluene emulsion and (B) water/petroleum ether emulsion.

**Table S1.** Sample names, starting composition proportions, and physical properties of DCPAs.

| Sample | TA (mol) | DETA (mol) | TREN (mol) | Solute  concentration (mg/ml) | ρ (mg/cm3^)[a]^ | LSR (%)^[b]^ | S_BET_ (m2/g)^[c]^ | Porosity (%)^[d]^ |
| --- | --- | --- | --- | --- | --- | --- | --- | --- |
| DCPA-**1** | 1 | 0.3 | 0.47 | 80 | 112.5±2.63 | 22.8±1.5 | 11.91 | 91.3 |
| DCPA-**2** | 1 | 0.3 | 0.47 | 100 | 121.4±11.2 | 15.4±2.6 | 11.71 | 90.7 |
| DCPA-**3** | 1 | 0.3 | 0.47 | 120 | 119.6±5.71 | 10.5±2.8 | 13.45 | 90.9 |
| recycled DCPA | — | — | — | 110 | 114.3±8.01 | 15.9±1.4 | 11.38 | 91.2 |

[a] Bulk density calculated as [weight]/[bulk volume]. [b] Linear shrinkage ratio (LSR) calculated as 100%×(1−[aerogel diameter]/[the corresponding wet gel diameter]). [c] Specific surface areas (SSAs) obtained using the Brunauer-Emmett-Teller (BET) method. [d] Porosity calculated as 100%×(1−[bulk density]/[skeleton density]).

Discussion

The thermal conductivities can be explained by the mathematical formula about the total thermal conductivity ($\lambda_{total}$) of aerogel that is the arithmetic sum of the radiative heat transfer coefficient ($\lambda_{r}$), solid thermal conductivity ($\lambda_{s}$), gas thermal conductivity ($\lambda_{g}$) (equation 1) [17-19]:

$$\lambda_{total}=\lambda_{r}+\lambda_{s}+\lambda_{g} (1)$$

In general, the $\lambda_{r}$ for aerogel is negligible in consideration of the minimal effect at room temperature because its value has a positive relation with temperature. Hence, the thermal conductivity of aerogel at room temperature is determined by the $\lambda_{s}$ and $\lambda_{g}$. On one hand, because the $\lambda_{s}$ for aerogel showed a strong density dependence, the low densities of our DCPAs would lead to low solid thermal conductivity [20]. On the other hand, the $\lambda_{g}$ obeys the following equation 2 [17, 18, 21]:

$$\lambda_{g}=\frac{\lambda_{g}^{0}\Pi}{1+{2\beta l_{mfp}}/{l_{cl}}} (2)$$

where $\lambda_{g}^{0}$ is the thermal conductivity of air, Π is the porosity of the aerogel, β is a constant about the energy transfer (approximately 2 for air in aerogel), $l_{mfp}$ is the mean free path of a gas molecule (air, 70 nm), and $l_{cl}$is the average pore diameter. According to equation 2, besides low density, the small pore size in a range from a few hundred nanometers to several micrometers that greatly decreased the gas thermal conductivity is another critical factor for the low thermal conductivities of our DCPAs.

**Caption for Supplementary Movie S1**

**Movie S1.** The continuous oil-water emulsion separation apparatus driven by a peristaltic pump

3. References

1. Kobayashi Y, Saito T and Isogai A. Aerogels with 3D ordered nanofiber skeletons of liquid-crystalline nanocellulose derivatives as tough and transparent insulators. *Angew Chem Int Ed* 2014; **53**(39): 10394-7.

2. Wu T, Dong J and De France K *et al.* Porous carbon frameworks with high CO_2_ capture capacity derived from hierarchical polyimide/zeolitic imidazolate frameworks composite aerogels. *Chem Eng J* 2020; **395**: 124927.

3. Teo N, Gu Z and Jana SC. Polyimide-based aerogel foams via emulsion-templating. *Polymer* 2018; **157**(21): 95-102.

4. Williams JC, Nguyen BN and McCorkle L *et al.* Highly porous, rigid-rod polyamide aerogels with superior mechanical properties and unusually high thermal conductivity. *ACS Appl Mater Interfaces* 2017; **9**(2): 1801-9.

5. Chen Y, Yu Z and Ye Y *et al.* Superelastic, hygroscopic, and ionic conducting cellulose nanofibril monoliths by 3d printing. *ACS Nano* 2021; **15**(1): 1869-79.

6. Jiang J, Oguzlu H and Jiang F. 3D printing of lightweight, super-strong yet flexible all-cellulose structure. *Chem Eng J*  2021; **405**: 126668.

7. Chen X, Liu H and Zheng Y *et al.* Highly compressible and robust polyimide/carbon nanotube composite aerogel for high-performance wearable pressure sensor. *ACS Appl Mater Interfaces* 2019; **11**(45): 42594-606.

8. Zhang X, Ni X and He M *et al.* A synergistic strategy for fabricating an ultralight and thermal insulating aramid nanofiber/polyimide aerogel. *Mater Chem Front* 2021; **5**: 804-816.

9. Zu G, Shimizu T and Kanamori K *et al.* Transparent, superflexible doubly cross-linked polyvinylpolymethylsiloxane aerogel superinsulators via ambient pressure drying. *ACS Nano* 2018; **12**(1): 521-32.

10. Qin Y, Peng Q and Ding Y *et al.* Lightweight, superelastic, and mechanically flexible graphene/polyimide nanocomposite foam for strain sensor application. *ACS Nano* 2015; **9**(9): 8933-41.

11. Yu Z-L, Yang N and Apostolopoulou-Kalkavoura V *et al.* Fire-retardant and thermally insulating phenolic-silica aerogels. *Angew Chem Int Ed* 2018; **57**(17): 4538-42.

12. Wang J, Liu D and Li Q *et al.* Lightweight, superelastic yet thermoconductive boron nitride nanocomposite aerogel for thermal energy regulation. *ACS Nano* 2019; **13**(7): 7860-70.

13. Schwan M and Ratke L. Flexibilisation of resorcinol–formaldehyde aerogels. *J Mater Chem A* 2013; **1**(43): 13462-8.

14. Xie C, Liu S and Zhang Q *et al.* Macroscopic-scale preparation of aramid nanofiber aerogel by modified freezing–drying method. *ACS Nano* 2021; **15**(6): 10000-9.

15. Wang L, Zhang M and Yang B *et al.* Highly compressible, thermally stable, light-weight, and robust aramid nanofibers/ti3alc2 mxene composite aerogel for sensitive pressure sensor. *ACS Nano* 2020; **14**(8): 10633-47.

16. Zhu J, Yang M and Emre A *et al.* Branched aramid nanofibers. *Angew Chem Int Ed* 2017; **56**(39): 11744-8.

17. Lu X, Arduini-Schuster MC and Kuhn J *et al.* Thermal conductivity of monolithic organic aerogels. *Science* 1992; **255**(5047): 971-2.

18. Wicklein B, Kocjan A and Salazar-Alvarez G *et al.* Thermally insulating and fire-retardant lightweight anisotropic foams based on nanocellulose and graphene oxide. *Nature Nanotechnol* 2015; **10**(3): 277-83.

19. Lee O-J, Lee K-H and Jin YT *et al.* Determination of mesopore size of aerogels from thermal conductivity measurements. *J Non-Cryst Solids* 2002; **298**(2): 287-92.

20. Qian Z, Yang M and Li R *et al.* Fire-Resistant, Ultralight, superelastic and thermal-insulated polybenzazole aerogels. *J Mater Chem A* 2018; **6**: 20769-77.

21. Rizvi A, Chu RKM and Park CB. Scalable fabrication of thermally insulating mechanically resilient hierarchically porous polymer foams. *ACS Appl Mater Interfaces* 2018; **10**: 38410-17.
